# The Impact of a Dedicated In-Hospital Vaccination Clinic on Adherence to Herpes Zoster Vaccination Among Immunocompromised and Frail Adults: Findings from an Italian Quasi-Experimental Study

**DOI:** 10.3390/vaccines14040306

**Published:** 2026-03-28

**Authors:** Alessandra De Pasquale, Adele Sarcone, Mariangela Cassadonte, Iole Camilla Iocca, Gabriella Di Giuseppe, Carmelo G. A. Nobile, Claudia Pileggi

**Affiliations:** 1 Department of Health Sciences, University of Catanzaro “Magna Græcia”, 88100 Catanzaro, Italy; alessandra.depasquale@studenti.unicz.it (A.D.P.); adele.sarcone@studenti.unicz.it (A.S.); mariangela.cassadonte@studenti.unicz.it (M.C.);; 2 Department of Experimental Medicine, University of Campania “Vanvitelli”, 80138 Naples, Italy; 3 Department of Clinical and Experimental Medicine, University of Catanzaro “Magna Græcia”, 88100 Catanzaro, Italy; nobile@unicz.it

**Keywords:** herpes zoster, in-hospital vaccination-dedicated clinic, immunocompromised patients, frailty, vaccination adherence

## Abstract

Background: Principal aims of this study were to evaluate, in frail immunocompromised patients, the willingness to receive the herpes zoster (HZ) vaccination and the impact of the in-hospital vaccination dedicated clinic on HZ vaccination adherence. Materials and methods: A non-randomized quasi-experimental intervention study was conducted in Southern Italy among immunocompromised patients. Patients enrolled in the in-hospital vaccination dedicated clinic were included in the experimental group, while the control group consisted of patients recruited in the General Practitioners clinics. Results: From April 2024 to March 2025, 369 patients were recruited, 183 of whom were included in the experimental group and 186 in the control group, The willingness to receive HZ vaccination was declared by 46.3% of subjects, and among those, 28.5% underwent vaccination. Logistic regression results showed that participants in the experimental group were significantly more likely to both declare willingness to receive vaccination (OR = 6.32; 95% CI 3.05–13.10) and to adhere to HZ vaccination (OR = 64.63; 95% CI 14.78–282.62). Conclusions: Our findings have shown that both willingness and adherence to HZ vaccination in immunocompromised subjects remain inadequate with respect to international targets and provide an effective model of integration between different professionals capable of promoting greater accessibility and adherence to HZ vaccination.

## 1. Introduction

Herpes zoster (HZ) is a viral infection determined by the reactivation of varicella-zoster virus (VZV). In infected subjects, VZV can remain latent in the cranial sensory ganglia and spinal cord and reactivate following immune impairment of any kind, such as older age, cancer or psychological stress. The main symptoms are characterized by vesicular rash with dermatomal distribution and pain in the same area. The most troublesome complication is postherpetic neuralgia (PHN), a chronic painful discomfort associated with a lower quality of life. This and the other possible complications such as ophthalmic, vascular, visceral and neurological manifestations or disseminated HZ occur more frequently in older and immunocompromised patients [1,2,3,4]. The Italian National Immunization Plan (NPVP) [5] offers free HZ vaccination to people older than 64 years and subjects with frail conditions. In respect to the target of 50% provided by the Italian Ministry of Health and despite the availability of a safety vaccination capable of preventing the most severe complications of HZ such as PHN [6], results of previous studies underlined a largely sub-optimal adherence in Italy, with HZ vaccine uptake ranging from 0.5% to 30% [7,8,9]. Among the most common causes for non-adherence to vaccination is the lack of indications from specialists, as highlighted by a recent survey conducted among physicians specialized in the care of patients affected by diseases that indicated HZ vaccination; only 42% of the specialists discuss HZ vaccination with eligible patients, and only 36% administer indicated vaccines to their patients [10]. Also, Wang et al. [11] in a recent meta-analysis analyzed the willingness to receive HZ vaccination worldwide, highlighting that just half of adults over 50 years were willing to receive the HZ vaccination. The most prominent barrier to HZ vaccination was the limited knowledge on HZ and the HZ vaccine, while one of the leading factors that influenced willingness to receive the HZ vaccine was found in getting a recommendation from a healthcare provider [11].

Principal aims of this study were to evaluate, in frail immunocompromised patients, (a) the willingness to receive the HZ vaccination; (b) the impact of the in-hospital vaccination dedicated clinic on HZ vaccination adherence; and (c) the determinants of these outcomes of interest.

## 2. Patients and Methods

### 2.1. Study Design and Population

A quasi-experimental non-randomized intervention study [12] was conducted from 12 April 2024 to 31 March 2025 enrolling two groups of patients: the first (experimental group) recruited at the in-hospital vaccination dedicated clinic of the Teaching Hospital of Catanzaro and the second (control group) at the clinics of the General Practitioners (GP_s_) of the Calabria Region (Italy). The choice to select the control group from GPs’ outpatient clinics was based on two considerations. First, similarly to what occurs in the hospital setting, frail patients visit their GP multiple times throughout the year. Second, within the organizational framework of the Italian National Health Service (NHS), GPs contribute to achieving the vaccination coverage targets established by the PNPV and are therefore able to administer vaccines directly in their clinics, particularly those against influenza, COVID-19, pneumococcal disease and, importantly, herpes zoster.

The study population included subjects with indications to receive HZ vaccine due to being older than 64 years, or in younger subjects, due to immunodeficiency conditions including primary immunodeficiency disorders due to active cancer, transplantation, asplenia, end stage kidney disease (ESKD) and immunosuppressive or immunomodulating medications. Also, frail subjects under 65 with chronic conditions such as cardiovascular, respiratory, gastrointestinal or renal conditions, diabetes or chronic liver disease were eligible for the HZ vaccination.

### 2.2. Data Collection

Data was collected by a questionnaire administered to eligible subjects who consented to participate in the study and through an interview conducted by appropriately trained physicians working at the in-hospital vaccination dedicated clinic. Experimental group patients consisted of subjects undergoing a periodic occupational surveillance visit, according to the Italian Legislative Degree 81/2008 [13], and of patients referred to the in-hospital vaccination dedicated clinic by specialists working in the same hospital in order to receive vaccination advice. In the experimental group, data was collected during the visit, while for the control group patients, the interview was performed in the waiting room of the GP clinics. In the in-hospital vaccination dedicated clinic, after the administration of the questionnaire to the patients, vaccination was offered and, in case of compliance, the dose was administered immediately, or an appointment was arranged. Patients belonging to the control group were recommended vaccination against HZ and, to verify vaccination compliance, a check was carried out through the Regional Vaccination Registry (GIAVA) [14] 6 months after enrollment in the study.

### 2.3. Ethics

The study protocol was approved by the Territorial Ethics Committee of Calabria Region (ID No. 121; 9 April 2024). All participants provided written informed consent before taking part in the study. This study was conducted in accordance with the Helsinki Declaration.

### 2.4. Survey Instruments

For each patient, a questionnaire (Supplementary Materials Questionnaire S1) was completed, divided into five sections. The first section was designed to collect demographic characteristics (sex, age, marital status occupations and education level); the second section collected information about the patient’s clinical status, focusing attention on the cause of the immune impairment (e.g., type and data of transplantation, primary kidney disease, type and duration of dialysis, type of cancer, immunosuppressant treatments, comorbidities, etc.). The third section collected data on the level of awareness of the population about HZ (having heard about HZ infections, symptoms, complications, prevention and eventually the knowledge about the vaccination). The fourth section focused on attitudes towards HZ infection and vaccination (risk of contracting infection, danger of the disease, usefulness of vaccination and dangers of vaccination). In the last part of the questionnaire, we asked each patient if they had already been vaccinated, if they wanted to do so and, in any case, why they answered yes or no.

### 2.5. Statistical Analysis

All statistical analyses were performed by STATA software program, version 18 (Stata Corporation, College Station, TX, USA). Descriptive data were reported as frequencies and percentages for categorical data, as well as means and standard deviations (SD) for continuous data. Demographic and baseline characteristics of the study population were compared using Chi square or Fisher’s exact tests for categorical variables and a *t*-test for continuous variables.

Multiple logistic regression models were developed to determine associations between the outcomes of interest and the independent variables explored. The two outcomes examined in this sample of immunocompromised and frail patients were willingness to receive HZ vaccination (Model 1) and adherence to HZ vaccination (Model 2). The model-building strategy proposed by Hosmer and Lemeshow [15] was used to select the independent variables included in the final multivariate logistic regression models.

In particular, the following independent variables were included in both models: gender (male = 0; female = 1); age (≤64 years = 0; 65–75 years = 1; ≥76 years = 2), study group (control group = 0; experimental group = 1), educational (primary and secondary school = 0; high school = 1; university degree = 2), working activity (retired = 0; employed = 1), cardiovascular diseases (no = 0; yes = 1); gastrointestinal diseases (no = 0; yes = 1); perceived health status (≤7 points = 0, ≥8 points = 1); numbers of NDCs (≤2 = 0; > 2 = 1); correct knowledge about HZ symptoms (no = 0; yes = 1); correct knowledge about HZ prevention measures (no = 0; yes = 1); concern about the risk of contracting HZ (poor/moderately = 0; concern/very concern = 1); perception of the danger of HZ infection (poor/moderately = 0; dangerous/very dangerous = 1); principal fonts of information about HZ vaccine (none = 0; healthcare workers = 1; other fonts = 2). Moreover, in Model 1 we also included the following: diabetes (no = 0; yes = 1); transplant patients (no = 0; yes = 1); correct knowledge about HZ infection risks (no = 0; yes = 1); correct knowledge about HZ complications (no = 0; yes = 1); correct knowledge about HZ vaccine indications (no = 0; yes = 1); perception of the usefulness of the vaccination against HZ (poor/moderately = 0; useful/very useful = 1); perception of the danger of HZ vaccination (poor/moderately = 0; dangerous/very dangerous = 1). Conversely, in the Model 2, we included the following independent variables: additional person in the household (none = 0; 1 = 1; ≥2 = 2), oncological diseases (no = 0; yes = 1), kidney diseases (no = 0; yes = 1), and autoimmune diseases (no = 0; yes = 1).

To reduce confounding arising from the quasi-experimental design and from baseline differences between participants recruited in the two settings (hospital vs. GPs), a propensity score was estimated to represent the probability of being recruited in the hospital setting based on socio-demographic, clinical, and perceptual characteristics. The propensity score was then included as a covariate in the logistic regression models to assess the independent effect of the setting on the two outcomes: willingness to receive HZ vaccination and actual adherence to HZ vaccination. Interaction terms between key independent variables were tested to assess potential effect modification, and multicollinearity was evaluated by examining variance inflation factors (VIFs) for all variables included in the multivariable models. The Odds Ratio (OR) and the related 95% Confidence Intervals (95% CI) were calculated. The level of statistical significance was set for values of *p* < 0.05.

To ensure the representativeness of the study population and the generalizability of the findings, a minimum sample size of 151 patients per group was calculated, assuming a 50% prevalence of willingness to receive HZ vaccination [16,17], with a 95% CI level and a 5% margin of error. To account for the expected unavailability or non-participation of some patients, an additional 15% was added, resulting in an increase of 55 participants and a total sample of 366 individuals. Similarly, for the outcome of adherence to HZ vaccination, a minimum of 59 patients per group was estimated based on an assumed prevalence of 25% [7,8,9], to which 18 additional patients (15%) were added, yielding a total sample of 136 participants.

## 3. Results

The study population was composed of 369 subjects divided in two groups, of whom 183 patients (49.6%) were enrolled at the in-hospital vaccination dedicated clinic (experimental group) and 186 patients (50.4%) were enrolled at the GPs clinics (control group).

The main characteristics of the study population are reported in Table 1. The age of participants ranged between 31 and 99 years (mean 68.3 ± 11.4 years); 51.3% were male and 73.1% were retired. Two-thirds (66.4%) of the included subjects suffered at least two comorbidities, with cardiovascular disease as the most frequent (61.5%), followed by diabetes (24.4%) and oncological diseases (20.3%).

Regarding knowledge (Table 2), it was found that only 14.6% of the sample had correct knowledge about HZ risk infection and less than 10% about HZ complications, while almost half of the patients (49.3%) correctly indicated HZ preventive measures, with a significantly higher prevalence in the experimental than in the control group (57.4% vs. 41.4%; χ^2^ = 9.423; *p* = 0.002). On the other hand, only 20.3% of the patients had correct knowledge about HZ vaccine indications, with no difference between study groups (22.4% vs. 18.3%; χ^2^ = 0.969; *p* = 0.325). In relation to the information about the HZ vaccine, 36.6% of the subjects did not know it, whereas among the remaining subgroups, 69.1% had acquired information by mass/social media or friends and relatives, and only 30.9% by healthcare professionals.

All enrolled patients were asked if they were willing to receive the HZ vaccine and, of the 171 (46.3%) who declared themselves available, only 105 patients, 28.5% of the total sample and 61.4% of those who declared themselves willing, joined the HZ vaccination campaign. Univariate analysis (Table 2) showed a significant difference between the experimental and control groups both in terms of willingness to receive the vaccination (64.5% vs. 28.5%; χ^2^ = 48.04; *p* < 0.0001) and adherence (83.1% vs. 13.2%; χ^2^ = 75.3; *p* < 0.0001). Among the reasons to refuse HZ vaccine, patients declared fear of vaccination adverse effects (36.3%), lack of confidence in vaccines (26%) and the perception of not being at risk of HZ infection (22.2%). On the contrary, adherence to the vaccination campaign was explained by 37.2% of patients with the belief they were at risk of infection and/or complications and by 35% with the advice received by physicians, mainly specialists (73.8%).

In multivariable logistic regression models (Table S1), the highest odds of being willing to receive the HZ vaccine (Model 1) were found in subjects with greater concern about the risk of contracting HZ infection (OR = 4.35; 95% CI 1.95–9.70), with low/moderate perception of the danger of the HZ vaccine (OR = 0.32; 95% CI = 0.14–0.73), and in those who perceived the vaccine as a very useful measure in preventing HZ (OR = 9.00; 95% CI 4.36–18.57). Furthermore, transplant patients (OR = 0.25; 95% 0.09–0.67) and those with diabetes (OR = 0.46; 95% CI 0.21–1.03) were significantly less likely to be willing to receive HZ vaccine, as were subjects who had received information about HZ vaccination from healthcare providers (OR = 0.23; 95% CI 0.09–0.55) and other sources, such as mass and social media or relatives (OR = 0.35 95% CI 0.17–0.70), compared with subjects who reported not receiving information. Finally, logistic regression results showed that participants in the experimental group were significantly more likely to both declare willingness to receive vaccination (OR = 6.32; 95% CI 3.05–13.10) and to adhere to HZ vaccination (Model 2) (OR = 64.63; 95% CI 14.78–282.62).

After adjustment, the propensity score remained statistically significant in Model 1 (OR = 3.15; 95% CI 1.28–7.77; *p* = 0.012), indicating that some baseline characteristics influencing the probability of being recruited in one of the two settings were also associated with participants’ stated willingness to be vaccinated. In contrast, in Model 2, evaluating actual HZ vaccination adherence, the propensity score was not significant (OR = 0.25; 95% CI 0.03–1.94; *p* = 0.179), whereas the recruitment setting remained strongly associated with vaccine adherence (OR = 49.8; 95% CI 11.1–221.8; *p* < 0.01), confirming the robustness of this association.

No statistically significant interactions were identified, and all VIF values were below commonly accepted thresholds.

## 4. Discussion

This quasi-experimental study sought to identify strategies to improve adherence to HZ vaccination in frail patients due to age or immunocompromised status. Once the willingness to receive the HZ vaccine was verified, a large part of subjects (46.3%) declared themselves willing; from this result, it can be concluded that most of the sample would not adhere to the HZ vaccination. Findings of previous studies highlighted worldwide variability in the willingness to consider vaccination against HZ, ranging from 17% in the Hong Kong population [18] to 85% in South Korea and Australia [11]. In the Italian context, a national-level survey reported willingness to receive the HZ vaccine strictly in line with our results, with 46% of the sample declaring they would consider getting it [8]. In another study performed in Southern Italy, the same geographical area as ours, only 26.6% of participants recruited in the waiting room of the GPs’ clinics had a positive attitude towards receiving HZ vaccination [19]. This result is very similar to the 28.5% prevalence we found in the control group patients recruited in GPs’ clinics.

The main reasons for declaring themselves against receiving the HZ vaccination were, for more than 60% of the subjects, the lack of confidence in vaccinations and the fear of possible vaccine side effects. In almost a quarter of the population, the lack of perception of the risk of contracting HZ disease was the reason for the patients’ unwillingness to receive HZ vaccination, and it can be easily explained by the very poor knowledge about HZ-related risks (14.6%) and its complications (9.8%).

The crucial role played by HCWs in ensuring vaccination compliance is well documented [11,20] and, certainly, the lack of HZ vaccination recommendation, received by less than one-third of the patients in our study sample, may explain the low rate of HZ vaccination willingness. It is possible to hypothesize that this lack of vaccine recommendation by HCWs, particularly physicians, may be perceived by patients as being driven by the physician’s doubts about the safety or efficacy of vaccinations, as highlighted by previous studies [21,22]. Also, according to a recent survey conducted among specialists with the aim to describe attitudes and practice toward HZ vaccination in their patients [10], only 42% of specialists reported initiating a conversation about HZ vaccination with their patients, and 36% stated that they administer vaccines. Insufficient time during visits was the main motivation provided by physicians, along with the contraindications in patients, although in this regard, the study results showed poor correct knowledge among specialists with respect to HZ vaccine indications [10]. This latter finding may explain the apparent contradiction in our study results, which show a significantly lower willingness to receive the HZ vaccine both among subjects who had received information from healthcare providers compared to those who had no specific information about the HZ vaccine and among high-intensity-care patients such as transplant recipients, diabetics, and patients with cardiovascular diseases. The pivotal role of healthcare providers in promoting patients’ willingness and adherence to vaccinations is such if their knowledge about vaccines is adequate. Conversely, insufficient or incomplete knowledge of specific topics related to vaccination, such that the healthcare provider is unable to provide adequate answers to the patient’s doubts, could undermine patients’ trust in medical advice and compromise their willingness to be vaccinated [23].

Our study data contribute to the debate about the interventions to enhance access to vaccinations among adults and, in particular, about the opportunity to implement in-hospital vaccination dedicated clinics as an effectiveness measure to increase vaccination uptake against HZ for frail patients. Indeed, we found that most patients who received HZ vaccination (83.1%) belonged to the experimental group recruited at the in-hospital vaccination dedicated clinic, with a significant difference compared to the control group, as shown in both univariate and multivariate statistical analyses. A previous study conducted by our group [24] to evaluate the impact of the presence of an in-hospital vaccination dedicated clinic on vaccination coverage rates among HCWs had already highlighted the effectiveness of this measure, showing a dramatic increase in uptake of all recommended vaccinations among this category of subjects who, despite extensive scientific evidence in this field, continue to be characterized by a high prevalence of vaccine hesitancy [25]. Similarly, the results of this study highlight the ability of the in-hospital vaccination clinic to promote vaccination compliance among the most vulnerable patients by breaking down several key barriers. First, the patient is vaccinated in the same facility where they undergo most health checks related to their underlying condition, without having to schedule additional visits related to vaccine administration. Second, in this organizational model, the specialist who treats the frail patient provides HZ vaccine recommendation but is relieved of the additional care burden resulting from the need for vaccination counselling. Indeed, vaccination counselling is provided by the physicians at the in-hospital vaccination dedicated clinic, who have specific training in this field and are able to mitigate the aforementioned issues related to the incomplete knowledge of other specialists in vaccination matters.

This study warrants acknowledgement of several limitations. First, it was a quasi-experimental study, and it is well-known that this study design is prone to bias, since participants are not randomized and groups may differ in several ways at baseline. However, the combined use of univariate analysis methods and multivariate regression is an approach that can mitigate the influence these differences could have on the outcome of interest. Furthermore, the wide 95% CI associated with the OR for increased adherence to HZ vaccination in the experimental group (14.78–282.62) reflects the relatively small size of the subgroup of vaccinated patients included in the analysis and the unbalanced distribution of events between groups. This pattern likely contributed to the instability of the estimate and may partially reflect sparse-data bias. Nevertheless, because the 95% CI does not include the null value of 1, the findings remain statistically incompatible with the hypothesis of no benefit from the intervention, as further confirmed by the associated *p*-value (<0.001) [26,27].

We also examined the distribution of events per variable (EPV) to assess the risk of model overfitting. In Model 2, a total of 171 observations were included, with 105 adherence events. The model incorporated 18 covariates, resulting in approximately 5.8 EPV. Although this value is slightly below the traditional rule-of-thumb of 10 EPV for logistic regression, it remains within a range considered acceptable in epidemiological studies when covariates are selected a priori based on theoretical and clinical relevance. In addition, multicollinearity diagnostics showed that all VIF values were below commonly accepted thresholds, supporting the stability of the regression estimates.

A propensity score adjustment was also performed to further explore the potential influence of baseline characteristics on group allocation. The propensity score was not statistically significant (*p* = 0.179), suggesting that the observed effect is unlikely to be explained by major baseline imbalances between the experimental and control groups. Finally, as with all context-specific interventions, caution is warranted when generalizing these findings to other settings.

From a methodological point of view, a strength of this study is the objective assessment of vaccination status, carried out through an electronic database rather than through self-reported data, which represents a significant limitation in many similar studies on this topic. Furthermore, it used a vaccination strategy for HZ in line with the most recent recommendations of scientific societies, which promote the integration of vaccination activities in hospital settings.

## 5. Conclusions

In conclusion, our findings have shown that despite specific recommendations [5], both willingness and adherence to HZ vaccination in immunocompromised and frail subjects remain inadequate respect to international targets [28] and provide an effective model of integration between different professionals capable of promoting greater accessibility and adherence to HZ vaccination. Further research aimed at confirming these results in other contexts and with the involvement of other vaccinations are strongly needed.

## Figures and Tables

**Table 1:** Demographic and baseline characteristics of the study participants in relation to willingness and uptake of HZ vaccination.

Characteristics	Total N (%)	Experimental Group N (%)	Control Group N (%)	Willingness to Receive HZ N (%)	Adherence to HZ Vaccination N (%)
	369	183 (49.6)	186 (50.4)	171 (46.3)	105 (61.4)
Socio-demographic profile Gender					
Male	189 (51.3)	106 (57.9)	83 (44.6)	80 (42.3)	50 (62.5)
Female	180 (48.7)	77 (42.1)	103 (55.4)	91 (50.6)	55 (60.4)
				χ^2^ = 2.509, 1 df, *p* = 0.113	χ^2^ = 0.076, 1 df, *p* = 0.782
Age, mean +/− SD	68.3 ± 11.4	64.2 ± 11.9	72.3 ± 9.4	66.6 ± 11.8	63.9 ± 12.3
≤64 years	109 (29.5)	78 (42.6)	31 (16.7)	58 (53.2)	44 (75.8)
65–75 years	150 (40.7)	72 (39.3)	78 (41.9)	70 (46.7)	42 (60)
≥76 years	110 (29.8)	33 (18.1)	77 (41.4)	43 (39.1)	19 (44.2)
				χ^2^ = 4.400, 2 df, *p* = 0.111	χ^2^ = 10.552, 2 df, *p* = 0.005
Marital status					
Married/cohabitant	113 (30.6)	55 (30.1)	58 (31.2)	52 (46)	34 (65.4)
Unmarried/widowed/separated/divorced	256 (69.4)	128 (69.9)	128 (69.8)	119 (46.5)	71 (59.6)
				χ^2^ = 0.006, 1 df, *p* = 0.934	χ^2^ = 0.499, 1 df, *p* = 0.48
Additional persons in the household					
None	63 (17.1)	29 (15.8)	34 (18.3)	33 (52.4)	22 (66.7)
1	208 (56.4)	102 (55.7)	106 (57)	90 (43.3)	47 (52.2)
≥2	98 (26.5)	52 (28.5)	46 (24.7)	48 (49)	36 (75)
				χ^2^ = 1.987, 2 df, *p* = 0.37	χ^2^ = 7.331, 2 df, *p* = 0.026
Educational level					
Primary and secondary school	149 (40.4)	55 (30.1)	94 (50.5)	50 (33.6)	24 (48)
High school	140 (37.9)	71 (38.8)	69 (37.1)	62 (44.3)	40 (64.5)
University Degree	80 (21.7)	57 (31.1)	23 (12.4)	59 (73.8)	41 (69.5)
				χ^2^ = 34.200, 2 df, *p* < 0.001	χ^2^ = 5.672, 2 df, *p* = 0.059
Working Activity					
Retired	272 (73.1)	144 (78.7)	155 (83.3)	115 (42.3)	62 (53.9)
Employed	97 (26.9)	39 (21.3)	31 (16.4)	56 (57.7)	43 (76.8)
				χ^2^ = 6.866, 1 df, *p* = 0.009	χ^2^ = 8.313, 1 df, *p* = 0.004
Medical conditions					
Number of NDCs					
≤2	245 (66.4)	121 (66.1)	124 (66.7)	121 (49.4)	79 (65.3)
>2	124 (33.6)	62 (33.9)	62 (33.3)	50 (40.3)	26 (52)
				χ^2^ = 2.72, 1 df, *p* = 0.099	χ^2^ = 2.636, 1 df, *p* = 0.104
Cardiovascular diseases					
No	142 (38.5)	88 (48.1)	54 (29)	76 (53.5)	53 (69.7)
Yes	227 (61.5)	95 (51.9)	132 (71)	95 (41.8)	52 (54.7)
				χ^2^ = 4.785, 1 df, *p* = 0.029	χ^2^ = 4.008, 1 df, *p* = 0.045
Diabetes					
No	279 (75.6)	152 (83.1)	127 (68.3)	146 (52.3)	91 (62.3)
Yes	90 (24.4)	31 (16.9)	59 (31.7)	25 (27.8)	14 (56)
				χ^2^ = 16.496, 1 df, *p* < 0.001	χ^2^ = 0.36, 1 df, *p* = 0.548
Gastrointestinal diseases					
No	312 (84.5)	133 (72.7)	179 (96.3)	138 (44.2)	74 (53.6)
Yes	57 (15.5)	50 (27.3)	7 (3.7)	33 (57.9)	31 (93.9)
				χ^2^ = 3.618, 1 df, *p* = 0.057	Fischer exact *p* < 0.001
Metabolic diseases					
No	312 (38.5)	152 (83.1)	160 (86.1)	148 (47.4)	90 (60.8)
Yes	57 (15.5)	31 (16.9)	26 (13.9)	23 (40.3)	15 (65.2)
				χ^2^ = 0.972, 1 df, *p* = 0.324	χ^2^ = 0.163, 1 df, *p* = 0.686
Kidney diseases					
No	302 (81.8)	124 (67.8)	178 (95.7)	142 (47)	84 (59.1)
Yes	67 (18.2)	59 (32.2)	8 (4.3)	29 (43.3)	21 (72.4)
				χ^2^ = 0.307, 1 df, *p* = 0.579	χ^2^ = 1.786, 1 df, *p* = 0.181
Oncological diseases					
No	294 (79.7)	153 (83.6)	141 (75.8)	138 (47)	89 (64.5)
Yes	75 (20.3)	30 (16.4)	45 (24.2)	33 (44)	16 (48.5)
				χ^2^ = 0.207, 1 df, *p* = 0.649	χ^2^ = 2.879, 1 df, *p* = 0.09
Respiratory diseases					
No	343 (92.9)	177 (96.7)	166 (89.2)	160 (46.6)	100 (62.5)
Yes	26 (7.1)	6 (3.3)	20 (10.8)	11 (42.3)	5 (45.5)
				χ^2^ = 0.183, 1 df, *p* = 0.669	Fischer exact *p* = 0.34
Autoimmune diseases					
No	296 (80.2)	158 (86.3)	138 (74.2)	137 (44.2)	89 (65)
Yes	73 (19.8)	25 (13.7)	48 (25.8)	34 (57.9)	16 (47)
				χ^2^ = 0.002, 1 df, *p* = 0.964	χ^2^ = 3.684, 1 df, *p* = 0.055
Transplant patients					
No	320 (86.7)	137 (74.9)	183 (98.4)	154 (48.1)	95 (61.7)
Yes	49 (13.3)	46 (25.1)	3 (1.6)	17 (34.7)	10 (58.8)
				χ^2^ = 0.082, 1 df, *p* = 0.079	χ^2^ = 0.053, 1 df, *p* = 0.818
*Liver* ^a^	*29 (59.2)*	*26 (56.5)*	*3 (100)*	*8 (4.7)*	*5 (62.5)*
*Kidney* ^a^	*19 (38.8)*	*19 (41.3)*	*0 (0)*	*8 (4.7)*	*4 (50)*
*Bone* ^a^	*1 (2)*	*1 (2.2)*	*0 (0)*	*1 (0.6)*	*1 (100)*
Perceived health status					
Poor/Fair/Good (≤7 points)	243 (65.9)	104 (56.8)	139 (74.7)	101 (41.7)	56 (55.4)
Very good/Excellent (≥8 points)	126 (34.1)	79 (43.2)	47 (25.3)	70 (55.6)	49 (70)
				χ^2^ = 6.532, 1 df, *p* = 0.011	χ^2^ = 3.695, 1 df, *p* = 0.055

SD: Standard deviation. ^a^ Only among transplant recipients.

**Table 2:** Knowledge and attitudes regarding HZ infection and vaccination among study participants in relation to willingness and uptake of HZ vaccination.

	Total N (%)	Experimental Group N (%)	Control Group N (%)	Willingness to Receive HZ N (%)	Adherence to HZ Vaccination N (%)
	369	183 (49.6)	186 (50.4)	171 (46.3)	105 (61.4)
Knowledge about HZ infection					
Correct knowledge about HZ infection risk (at least 2 correct answer)					
No	315 (85.4)	153 (83.6)	162 (87.1)	132 (41.9)	80 (60.6)
Yes	54 (14.6)	30 (16.4)	24 (12.9)	39 (72.2)	25 (64.1)
				χ^2^ = 17.039, 1 df, *p* < 0.001	χ^2^ = 0.155, 1 df, *p* = 0.694
Correct knowledge about HZ symptoms (at least 3 correct answer)					
No	265 (71.8)	127 (69.4)	138 (74.2)	114 (43)	66 (57.9)
Yes	104 (28.2)	56 (30.6)	48 (25.8)	57 (54.8)	39 (68.4)
				χ^2^ = 4.174, 1 df, *p* = 0.041	χ^2^ = 1.776, 1 df, *p* = 0.183
Correct knowledge about HZ complications (at least 2 correct answer)					
No	333 (90.2)	163 (89.1)	170 (91.4)	144 (43.2)	91 (63.2)
Yes	36 (9.8)	20 (10.9)	16 (8.6)	27 (75)	14 (51.8)
				χ^2^ = 13.176, 1 df, *p* < 0.001	χ^2^ = 1.234, 1 df, *p* = 0.267
Correct knowledge about HZ measures of prevention (at least 2 correct answer)					
No	187 (50.7)	78 (42.6)	109 (58.6)	60 (32.1)	28 (46.7)
Yes	182 (49.3)	105 (57.4)	77 (41.4)	111 (61)	77 (69.4)
				χ^2^ = 30.986, 1 df, *p* < 0.001	χ^2^ = 8.470, 1 df, *p* = 0.004
Correct knowledge about HZ vaccine indication (at least 1 correct answer)					
No	294 (79.7)	142 (77.6)	152 (81.7)	124 (42.2)	75 (60.5)
Yes	75 (20.3)	41 (22.4)	34 (18.3)	47 (62.7)	30 (63.8)
				χ^2^ = 10.089, 1 df, *p* = 0.001	χ^2^ = 0.161, 1 df, *p* = 0.688
Perception of the risk of contracting HZ and attitudes to vaccines					
Concern about the risks of contracting HZ					
Poor/moderately	277 (75.1)	135 (73.8)	142 (76.3)	106 (38.3)	68 (64.1)
Concern/very concern	92 (24.9)	48 (26.2)	44 (23.7)	65 (70.6)	37 (56.9)
				χ^2^ = 31.357, 2 df, *p* < 0.001	χ^2^ = 3.000, 2 df, *p* = 0.223
Concern about dangerousness of the HZ disease					
Poor/moderately	218 (59.1)	106 (57.9)	112 (60.2)	77 (35.3)	51 (66.2)
Dangerous/very dangerous	151 (40.9)	77 (42.1)	74 (39.8)	94 (62.2)	54 (57.4)
				χ^2^ = 31.004, 2 df, *p* < 0.001	χ^2^ = 1.607, 2 df, *p* = 0.448
Perception of the usefulness of HZ vaccination					
Poor/moderately	181 (49.1)	72 (39.3)	109 (58.6)	39 (21.5)	24 (61.5)
Useful/very useful	188 (50.9)	111 (60.6)	77 (41.4)	132 (70.2)	81 (61.4)
				χ^2^ = 101.927, 2 df, *p* < 0.001	χ^2^ = 0.036, 2 df, *p* = 0.982
Perception of dangerousness of HZ vaccination					
Poor/moderately	290 (78.6)	149 (81.4)	141 (75.8)	150 (51.7)	91 (60.7)
Dangerous/very dangerous	79 (21.4)	34 (18.6)	45 (24.2)	21 (26.6)	14 (66.7)
				χ^2^ = 35.302, 2 df, *p* < 0.001	χ^2^ = 0.308, 2 df, *p* = 0.857
Previous episodes of HZ					
No/Do not remember	323 (87.5)	159 (86.9)	164 (88.2)	148 (45.8)	93 (62.8)
Yes	46 (12.5)	24 (13.1)	22 (11.8)	23 (50)	12 (52.2)
				χ^2^ = 0.282, 1 df, *p* = 0.595	χ^2^ = 0.955, 1 df, *p* = 0.328
*Head and neck* ^a^	*9 (19.6)*	*7 (29.2)*	*2 (9.1)*	*7 (77.8)*	*4 (57.1)*
*Upper trunk* ^a^	*12 (26.1)*	*6 (25.0)*	*6 (27.3)*	*3 (25)*	*0 (0)*
*Upper limbs* ^a^	*5 (10.9)*	*3 (12.5)*	*2 (9.1)*	*1 (20)*	*0 (0)*
*Lower limbs* ^a^	*5 (10.9)*	*2 (8.3)*	*3 (13.1)*	*4 (80)*	*3 (75)*
*Lower trunk* ^a^	*15 (32.6)*	*6 (25.0)*	*9 (40.9)*	*8 (53.3)*	*5 (62.5)*
Fonts of information about HZ vaccine					
None	135 (36.6)	86 (47)	49 (26.3)	95 (70.4)	66 (69.5)
Healthcare workers	92 (24.9)	40 (21.9)	52 (28)	20 (21.7)	10 (50)
Other fonts (mass media, journals, friends or familiars)	142 (38.5)	57 (31.1)	85 (45.7)	56 (39.4)	29 (51.8)
				χ^2^ = 56.463, 2 df, *p* < 0.001	χ^2^ = 0.288,2 df, *p* = 0.23

^a^ Only among those who reported previous episodes of HZ.

## Data Availability

All data generated or analyzed during the study are included in this published article. The dataset used during the current study is available from the corresponding author on reasonable request.

## Supplementary Materials

The following supporting information can be downloaded at: https://www.mdpi.com/article/10.3390/vaccines14040306/s1, Table S1: Multiple stepwise logistic regression analysis results.
