# Supplementary material for: The Impact of a Dedicated In-Hospital Vaccination Clinic on Adherence to Herpes Zoster Vaccination Among Immunocompromised and Frail Adults: Findings from an Italian Quasi-Experimental Study
Source: Vaccines (Basel). 2026 Mar 28;14(4):306. doi: 10.3390/vaccines14040306 (PMC13119745; doi:10.3390/vaccines14040306)
Supplement: Supplementary file 1 [file vaccines-14-00306-s001.zip › vaccines-4177240-supplementary-revised.pdf]

"MAGNA GRÆCIA" UNIVERSITY OF CATANZARO
SCHOOL OF MEDICINE AND SURGERY
CHAIR OF HYGIENE

QUESTIONNAIRE
Willingness and adherence to Herpes zoster vaccination among immunocompromised and frail subjects

The Vaccination Center of the Teaching Hospital of Catanzaro has promoted this survey to assess the knowledge, attitudes, and behaviors of immunocompromised and frail patients regarding Herpes zoster vaccination. THE QUESTIONNAIRE IS ANONYMOUS. In accordance with Legislative Decree 101/2018 (Personal Data Protection Code), the analyses conducted on the collected data will not involve any nominative disclosure. The information provided in the questionnaire does not allow for your personal identification; therefore, you may feel free to answer the questions.

□ UMG Vaccination Clinic   □ General Practitioner Clinic

Section A. SOCIO-DEMOGRAPHIC INFORMATION

In this section there are questions about sociodemographic characteristics.

A1. What is your gender? □ Male □ Female

A2. What is your marital status? □ Single □ Married □ Cohabiting □ Separated □ Other

A3. What is the highest level of education you have completed?

□ None □ Primary School □ Lower Secondary School □ Upper Secondary school □ University Degree in ________________________

A4. What is your current occupation?________________________

A5. Excluding yourself, how many people do you live with?_______________

Section B. CLINICAL INFORMATION

In this section there are questions about personal clinical history.

B1. Please list the diseases you suffer from and specify how long you have had them.

PATHOLOGY	DURATION

B2. On a scale from 1 to 10, how would you rate your current health status?

1 2 3 4 5 6 7 8 9 10

Section C. KNOWLEDGE

In this section there are questions about knowledge regarding Shingles (Herpes zoster) infection and vaccination.

C1. Shingles (Herpes zoster) is a disease characterized by blisters, vesicles, itching and/or pain. Have you ever heard of it?

□ No □ Yes, from whom? □ Family □ Friends □ TV, newspapers, magazines □ Internet/social media □ Pharmacists □ Physicians (GP or specialist) ____________________ □ Other ____________________

(If the answer to C1 question is No, skip directly to C7 question)

C2. Which factors may favor the onset of Shingles? (multiple answers possible)
□ I do not know □ Older age □ Stress □ Vitamin deficiency □ Immunosuppressive therapy □ Depression □ Diabetes □ Cancer □ Other ______________________

C3. Which symptoms may occur with Shingles? (multiple answers possible)
□ I do not know □ Blisters/vesicles □ Burning/pain □ Itching □ Ocular manifestations □ Headache □ Fever □ General malaise □ Loss of skin sensitivity □ Other ______________

C4. What complications may result from Shingles? (multiple answers possible)
□ I do not know □ Chronic pain □ Ocular complications □ Disseminated Herpes Zoster □ Other ____________________

C5. Can chronic pain associated with Shingles affect your normal daily activities?
□ I do not know □ Slightly □ Moderately □ Significantly □ Very significantly

C6. How can Shingles be prevented? (multiple answers possible)
□ I do not know □ Hand washing □ Avoid contact with infected individuals □ Avoid contact with clothing of infected individuals □ Avoid contact with personal items of infected individuals □ Vaccination □ Other ______________________________________

C7. In Italy, a vaccine is available to prevent Shingles. Have you ever heard about it?
□ No □ Yes, from whom? □ Family □ Friends □ TV, newspapers, magazines □ Internet/social media □ Pharmacists □ Physicians (GP or specialist) _____________________ □ Other _____________________

C8. For whom is the vaccine recommended? (multiple answers possible):
□ I do not know □ Age > 65 years □ Age ≥ 50 years □ age ≥ 50 years with risk factors □ age< 50 years

C9. How many doses are administered?
□ I do not know □ 1 □ 2

Section D. ATTITUDES

In this section there are questions about the awareness of the risk of contracting Shingles (Herpes zoster) and/or to develop complications connected and attitudes in theme of vaccination anti Herpes zoster.

D1. How concerned are you about contracting Shingles? *(1 = not concerned; 10 = very concerned)*

NOT CONCERNED 1 2 3 4 5 6 7 8 9 10 VERY CONCERNED

D2. How dangerous do you think Shingles is? *(1 = not dangerous; 10 = very dangerous)*

NOT DANGEROUS 1 2 3 4 5 6 7 8 9 10 VERY DANGEROUS

D3. How useful do you think the Herpes Zoster vaccine is? *(1 = not useful; 10 = very useful)*

NOT USEFUL 1 2 3 4 5 6 7 8 9 10 VERY USEFUL

D4. How dangerous do you think the Herpes Zoster vaccine is? *(1 = not dangerous; 10 = very dangerous)*

NOT DANGEROUS 1 2 3 4 5 6 7 8 9 10 VERY DANGEROUS

Section E. BEHAVIORS

In this section there are questions about behaviours related to Herpes zoster vaccination.

E1. Have you had chickenpox in the past? □ No □ Yes □ I do not remember

E2. If yes, at what age did you have chickenpox? ______ □ I do not remember

E3. Have you been vaccinated against chickenpox? □ No □ Yes □ I do not remember

E4. If yes, at what age? ______ □ I do not remember

E5. Have you had Shingles (Herpes zoster)? □ No □ Yes □ I do not remember

E6. If yes, at what age did you have Shingles? ______ □ I do not remember

E7. If yes, where did the vesicles/pain appear? ______________________________
□ I do not remember

E8. Have you been vaccinated against Shingles (Herpes Zoster)?

□ No, why? (multiple answers possible)

□ I was not aware of the vaccination
□ It is not useful
□ I do not feel at risk
□ Fear of vaccination side effects
□ Not recommended by GP
□ Not recommended by specialist:
□ I do not believe in vaccinations
□ Other

□ Yes (Thank you. You have now completed the questionnaire)

E9. Would you like to get vaccinated against Shingles (Herpes zoster)??

□ No, why? (multiple answers possible)	□ Yes, why? (multiple answers possible)
□ It is not useful	□ It is useful
□ I do not feel at risk	□ I feel at risk
□ Fear of vaccination side effects	□ Fear of complications of the infection
□ Not recommended by GP	□ Recommended by GP
□ Not recommended by specialist:	□ Recommended by specialist:
□ Other	□ Other

⇩

Save the date

Date 1st dose ____________________

Date 2nd dose____________________
