# Supplementary material for: The Impact of a Dedicated In-Hospital Vaccination Clinic on Adherence to Herpes Zoster Vaccination Among Immunocompromised and Frail Adults: Findings from an Italian Quasi-Experimental Study
Source: Vaccines (Basel). 2026 Mar 28;14(4):306. doi: 10.3390/vaccines14040306 (PMC13119745; doi:10.3390/vaccines14040306)
Supplement: Supplementary file 1 [file vaccines-14-00306-s001.zip › vaccines-4177240-supplementary.pdf]

Table S1. Multiple stepwise logistic regression analysis results.

Variable	OR	SE	95% CI	*p* value
**Model 1: Willingness to receive HZ**				
Log-likelihood=-149.69, Chi-square=210.18, p=0.0000, N of obs=369				
**Study group**				
Experimental group	6.32	2.35	3.05-13.10	***< 0.001***
(Control group as reference)				
**Concern about the risk of HZ infection**				
Concern/very concern	4.35	1.77	1.95-9.70	***< 0.001***
(Poor/moderately as reference)				
**Perception of the usefulness of HZ vaccination**				
Useful/very useful	9.00	3.32	4.36-18.57	***< 0.001***
(Poor/moderately as reference)				
**Principal fonts of information about HZ vaccine**				
Healthcare workers	0.23	0.10	0.09-0.55	***0.001***
Other fonts (mass media, journals, friends, familiar members)	0.35	0.12	0.17-0.69	***0.003***
(None as reference)				
**Transplant patients**				
Yes	0.25	0.12	0.09-0.67	***0.006***
(No as reference)				
**Perception of dangerousness of HZ vaccination**				
Dangerous/very dangerous	0.32	0.13	0.14-0.73	***0.007***
(Poor/moderately as reference)				
**Correct knowledge about HZ symptoms**				
Yes	0.45	0.18	0.20-1.00	0.052
(No as reference)				
**Diabetes**				
Yes	0.46	0.18	0.21-1.03	0.061
(No as reference)				
**Gender**				
Female	1.64	0.52	0.87 – 3.07	0.122
(Male as reference)				
**Concern about dangerousness of the HZ disease**				
Dangerous/very dangerous	0.60	0.23	0.28-1.27	0.185
(Poor/moderately as reference)				
**Educational level**				
High school	1.16	0.38	0.60-2.23	0.657
University Degree	1.50	0.71	0.58-3.84	0.393
(Primary and secondary school as reference)				
**Correct knowledge about HZ infection risk**				
Yes	1.48	0.76	0.53-4.08	0.445
(No as reference)				
**Working Activity**				
Employed	1.32	0.54	0.58-2.98	0.500
(Retired as reference)				
**Correct knowledge about HZ complications**				
Yes	1.47	0.91	0.43-4.95	0.533
(No as reference)				
**Correct knowledge about HZ measures of prevention**				
Yes<br>(No as reference)	1.20	0.40	0.63-2.31	0.566

Variable	OR	SE	95% CI	*p* value
**Gastrointestinal diseases**				
Yes	0.83	0.40	0.32-2.16	0.712
(No as reference)				
**Number of NDCs**				
>2	0.90	0.33	0.43-1.85	0.780
(≤2 as reference)				
**Metabolic diseases**				
Yes	0.91	0.32	0.45-1.82	0.790
(No as reference)				
**Perceived health status**				
Very good/ Excellent (≥ 8 points)	0.97	0.34	0.48-1.96	0.899
(Poor/Fair/Good (≤ 7 points) as reference)				
**Age, mean +/- SD**				
65-75 years	0.95	0.38	0.43-2.11	0.906
≥ 76 years	1.05	0.49	0.42-2.65	0.906
(≤ 64 years as reference)				
**Correct knowledge about HZ vaccine indication**				
Yes	1.01	0.39	0.46 – 2.19	0.972
(No as reference)				

Variable	OR	SE	95% CI	*p* value
**Model 2: Adherence to HZ vaccination**				
Log-likelihood=-62.57, Chi-square=102.93, p=0.0000, N of obs=171				
**Study group**				
Experimental group (Control group as reference)	64.63	48.65	14.78-282.62	***< 0.001***
**Additional persons in the household**				
1	0.25	0.18	0.06-1.06	0.061
≥2	1.88	1.63	0.34-10.30	0.463
(None as reference)				
**Educational level**				
High school	2.26	1.40	0.67-7.61	0.187
University Degree	1.40	0.94	0.37-5.24	0.613
(Primary and secondary school as reference)				
**Perceived health status**				
Very good/ Excellent (≥ 8 points)	0.50	0.29	0.16-1.57	0.237
(Poor/Fair/Good (≤ 7 points) as reference)				
**Oncological diseases**				
Yes (No as reference)	0.51	0.30	0.16-1.66	0.269
**Gastrointestinal diseases**				
Yes	2.49	2.40	0.37-16.47	0.342
(No as reference)				
**Age, mean +/- SD**				
65-75 years	1.65	1.11	0.44-6.17	0.451
≥ 76 years	1.87	1.51	0.38-9.14	0.437
(≤ 64 years as reference)				
**Kidney diseases**				
Yes	0.63	0.45	0.15-2.58	0.521
(No as reference)				
**Metabolic diseases**				
Yes	0.73	0.41	0.24-2.20	0.578

Variable	OR	SE	95% CI	*p* value
(No as reference)				
**Number of NDCs**				
>2	0.70	0.44	0.20-2.45	0.588
(≤2 as reference)				
**Fonts of information about HZ vaccine**				
Other fonts (mass media, journals, friends or familiars)	0.80	0.40	0.30-2.14	0.666
(None as reference)				
**Correct knowledge about HZ symptoms**				
Yes	1.24	0.70	0.41-3.74	0.701
(No as reference)				
**Working Activity**				
Employed	1.28	0.86	0.34-4.78	0.705
(Retired as reference)				
**Correct knowledge about HZ measures of prevention**				
Yes	1.21	0.61	0.44-3.28	0.706
(No as reference)				
**Autoimmune diseases**				
Yes	1.08	0.75	0.28-4.21	0.903
(No as reference)				
**Gender**				
Female	1.01	0.51	0.37-2.75	0.976
(Male as reference)				
**Concern about dangerousness of the HZ disease**				
Dangerous/very dangerous	0.99	0.49	0.37-2.63	0.997
(Poor/moderately as reference)				

SE: Standard error
